# Trachomatous scarring among children in a formerly hyper-endemic district of Tanzania

**DOI:** 10.1371/journal.pntd.0006085

**Published:** 2017-12-12

**Authors:** Jacob T. Cox, Harran Mkocha, Beatriz Munoz, Sheila K. West

**Affiliations:** 1 Dana Center for Preventive Ophthalmology, Wilmer Eye Institute, Johns Hopkins Hospital, Baltimore, Maryland, United States of America; 2 Kongwa Trachoma Project, Kongwa, Tanzania; RTI International, UNITED STATES

## Abstract

**Background:**

Associations between repeated ocular infections with *Chlamydia trachomatis* in childhood and conjunctival scarring in adulthood are well established. Trachomatous scarring (TS) in children has also been observed in hyper-endemic areas, but data are scant regarding childhood scarring in areas where trachoma has been reduced to hypo-endemic levels.

**Methods/Principle findings:**

In this cross-sectional study, a random sample of children, ages 1–9 years, were selected from 38 communities in the formerly hyper-endemic district of Kongwa, Tanzania. Each participant received an ocular examination and eye-swab test for *C*. *trachomatis* infection. Conjunctival photographs were taken and analyzed at 5x magnification to determine scarring presence and severity. Community-level case clustering was assessed using intra-class correlation coefficients, and associations between TS presence and demographic/clinical factors were assessed using contingency table analyses. 1,496 children (78% of eligible) participated in this study. The mean age was 5.5 years and 51% were female. Scarring prevalence was 2.1% (95% CI: 1.5%– 3.0%). The prevalence of follicular trachoma and ocular *C*. *trachomatis* infection were 3.2% and 6.5%, respectively. Most TS cases (68.7%) fell into the mildest category, grade S1. 18.7% were grade S2; 12.6% were grade S3. No significant associations were seen between TS presence and age, sex, follicular trachoma, or active ocular *C*. *trachomatis* infection (*p*-values: 0.14, 0.48, 0.27, 0.15, respectively). Thirty communities (78.9%) had 0–1 TS cases, and the most seen in any single community was four cases. Three years ago, follicular trachoma prevalence averaged 4.9% in communities with 0–1 TS cases, but 7.6% in communities with 2–4 TS cases (*p*-value: 0.08).

**Conclusions:**

In this formerly hyper-endemic district of Tanzania, TS was rare in 1–9 year-olds and usually mild when present. Communities with higher rates of follicular trachoma in the past were more likely to have ≥2 cases of scarring, but the association was not statistically significant.

## Author summary

Trachoma is a disease in which the conjunctiva of the eye is repeatedly infected by the intracellular organism *Chlamydia trachomatis*. This can cause scarring of the eyelid’s inner surface, which in turn can cause the eyelashes to turn inwards, scratching the eye and leading to blindness. This scarring process is associated with age and usually does not present until adulthood. However, in communities with very high trachoma rates, it has also been reported in children. There are scant data addressing the presence and nature of such scarring among children in areas where elimination efforts have reduced trachoma from high to low prevalence. In this study, we found that even though interventions in Kongwa, Tanzania, have successfully reduced trachoma levels in recent years, 1–9 year-olds can still be found with eyelid scarring. However, this scarring is rare and generally mild. Eyelid scarring from trachoma is known to increase with age, but interestingly, that was not seen in this cohort. This suggests that not only have interventions reduced eyelid scarring in children to low levels, but that this protective affect may be sustained into adolescence and adulthood.

## Introduction

Trachoma results from repeated ocular infection with *Chlamydia trachomatis* and is the leading infectious cause of blindness worldwide. [1] While the conjunctival inflammation associated with active infection is predominantly seen before 10 years of age, the resultant conjunctival scarring usually presents in adulthood and is associated with age both in prevalence and severity. [2–4] Nevertheless, conjunctival scarring has been documented in children in regions where trachoma prevalence is 30% or higher, and in one longitudinal study, children who were observed to have severe trachomatous inflammation on multiple occasions were most at risk of having scarring. [2,5,6] There is no animal reservoir for *C*. *trachomatis*, and transmission is from person to person through infected ocular and nasal secretions.

The World Health Organization has endorsed the SAFE strategy for trachoma control, which consists of Surgery for end-stage trichiasis, and Antibiotic use, Facial cleanliness, and Environmental improvements to reduce the active, infectious stage of the disease. At present, there is no direct intervention to decrease scarring, except to reduce the frequency/severity of the repeated episodes of infection.[7] In the context of trachoma-reduction programs like GET 2020 (Global Elimination of Blinding Trachoma by 2020 Initiative), trachoma prevalence has decreased significantly in a number of areas that were once hyper-endemic. [8–10] Yet there is a lack of data describing the prevalence or severity of trachomatous scarring (TS) in children in these formerly hyper-endemic areas.

This study’s primary objective is to determine the prevalence of TS among children, ages 1–9 years, in the formerly hyper-endemic district of Kongwa, Tanzania. Secondary aims include determining the severity of TS, assessing associations between TS and various demographic or clinical factors, and evaluating community-level case clustering.

## Methods

### Ethical statement

This study complied fully with the Declaration of Helsinki and the guardians of all participants provided informed, written consent. The Johns Hopkins Institutional Review Board and the National Institute of Medical Research of the United Republic of Tanzania approved of this study.

### Population

Kongwa district has been known for its high trachoma prevalence and scarring risk in children, as documented in previous studies. [6,11] In 1986, the prevalence of trachoma in children ages 1–7 years (pre-school age) was 60%. By 2010, Kongwa district had an overall prevalence in children of 17%.[11,12] Recent data suggest that trachoma has declined to less than 10% in children age 1 to 9 years in this district, suggesting there may be a declining risk of the scarring complications of trachoma. [13] Participants age 1–9 years were randomly selected based on a census conducted in 38 communities in Kongwa that were already involved in a separate study. [13] Fifty children were randomly selected from each community; two alternates were also randomly selected to ensure the goal of 50 children would be achieved. Data collection took place at central sites within participants’ villages.

### Clinical examination

Participants received an ocular examination of the right upper tarsal conjunctiva by a trained grader to assess for follicular trachoma using 2.5x loupe and a flashlight. The grader also collected an eye swab from the child’s right eye to assess for *C*. *trachomatis* infection using the Aptima Combo at Johns Hopkins International Chlamydia Laboratory. A 5% sample of “air swabs” was also collected to assess contamination. Digital photographs of each child’s right upper tarsal conjunctiva were captured (D40; Nikon, Tokyo, Japan). Two trained graders at Johns Hopkins Hospital examined these photographs at 5x magnification to determine scarring severity. They were masked to one another’s scores, and scores were determined using a validated, five-point scale developed by Wolle *et al*. [14] S1 is the presence of at least one linear scar of size 3 mm or less, with or without stellate scars, S2 is multiple lines of scarring of more than 3mm that occupy up to 1/8 of the upper tarsus; S3 is a pattern of scarring that occupies up to 1/3 of the eyelid and S4 is >90% of the tarsus obliterated by scarring. S0 is an eyelid that does not meet the criteria for S1 or worse. Any discordant grades were openly adjudicated with a senior grader. Before starting, inter-grader agreement was evaluated using 60 images from a separate dataset, and each grader was required to achieve a kappa 0.70 or greater relative to the senior grader.

### Statistical methods

Descriptive statistics were used to characterize the study population. Contingency table analyses were used to evaluate differences between participants and non-participants. The TS prevalence with exact 95% confidence intervals (95% CIs) is presented. The confidence interval was not adjusted for clustering, as there was no evidence for clustering by community. Due to the small number of TS cases, the data was collapsed from an ordinal scale to a simple presence or absence. Chi-square and Fisher’s exact testing were used, as appropriate, to assess for associations between TS presence and age, sex, follicular trachoma, or ocular *C*. *trachomatis* infection. To assess the community-level clustering of TS cases, the intra-class correlation coefficient and corresponding 95% CIs were estimated. Follicular trachoma prevalence rates three years prior to the TS assessment were compared between communities with zero or one TS case and communities with 2 or more cases based on medians and interquartile ranges using a Wilcoxson 2-sample test. All analyses were run using SAS 9.2 software (SAS Institute, Cary, NC). Data are available in supplementary file 1 (S1 Data). For the STROBE checklist, please see S2 Data.

## Results

**Fig 1:**
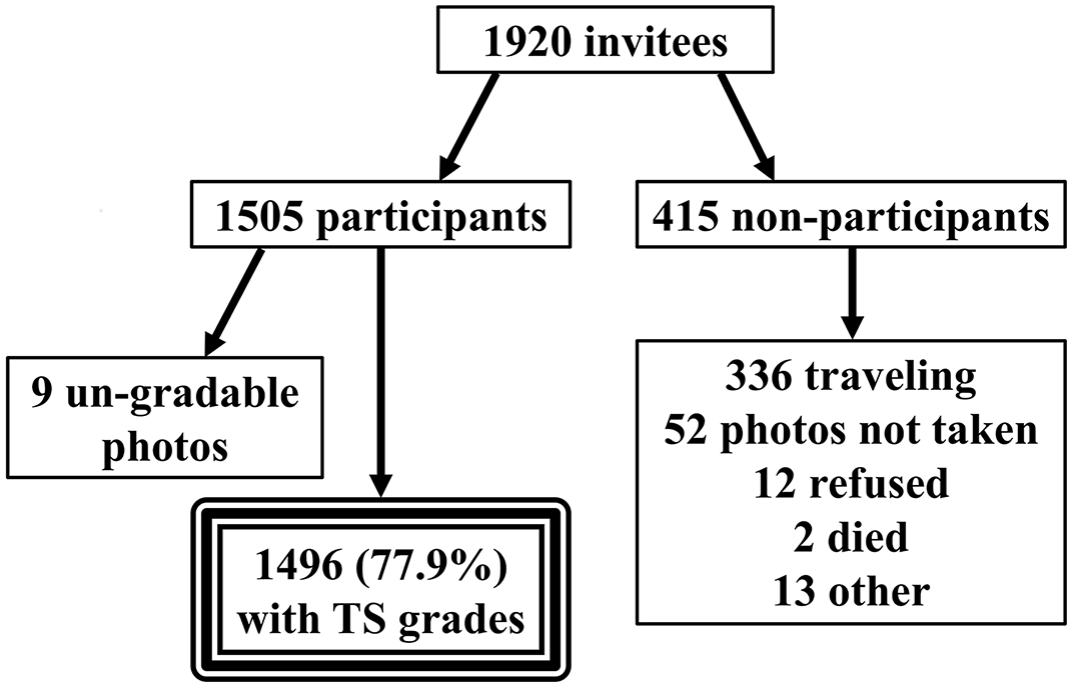
Schematic demonstrating process of participant inclusion and exclusion.

A total of 1,505 children (78.4% of invitees) participated in this study. (Fig 1) Of these, nine participants (0.6%) had un-gradable photographs and were excluded from subsequent analysis. As shown in Table 1, the only significant difference between participants and non-participants was the availability of a household latrine (*p*-value < 0.001). The remaining factors assessed showed no significant difference between participants and non-participants. None of the air controls for the test of infection demonstrated evidence of contamination.

**Table 1:** Comparison between participants and non-participants. Age, sex, and socio-economic status proxies are included because these are known to be associated with trachoma.

Characteristic	Participants (*n* = 1,505)	Non-participants (*n* = 415)	*p*-value
Age, mean ± SD	5.5 ± 2.6	5.4 ± 2.7	0.64
Sex, *n* (%)			
Male	737 (49.0)	218 (52.5)	0.20
Female	768 (51.0)	197 (47.5)	
Formal education of head of household, *n* (%)			
None	551 (36.6)	166 (40.0)	0.23
Some	954 (63.4)	249 (60.0)	
Latrine in household, *n* (%)			
No	303 (20.1)	125 (30.0)	0.001
Yes	1202 (79.9)	290 (70.0)	
Lives > 30 min. from water, *n* (%)			
No	691 (45.9)	180 (43.3)	0.35
Yes	814 (54.1)	235 (56.7)	
Bicycle in household, *n* (%)			
No	751 (49.9)	212 (51.2)	0.55
Yes	754 (50.1)	203 (48.8)	

**Fig 2:**
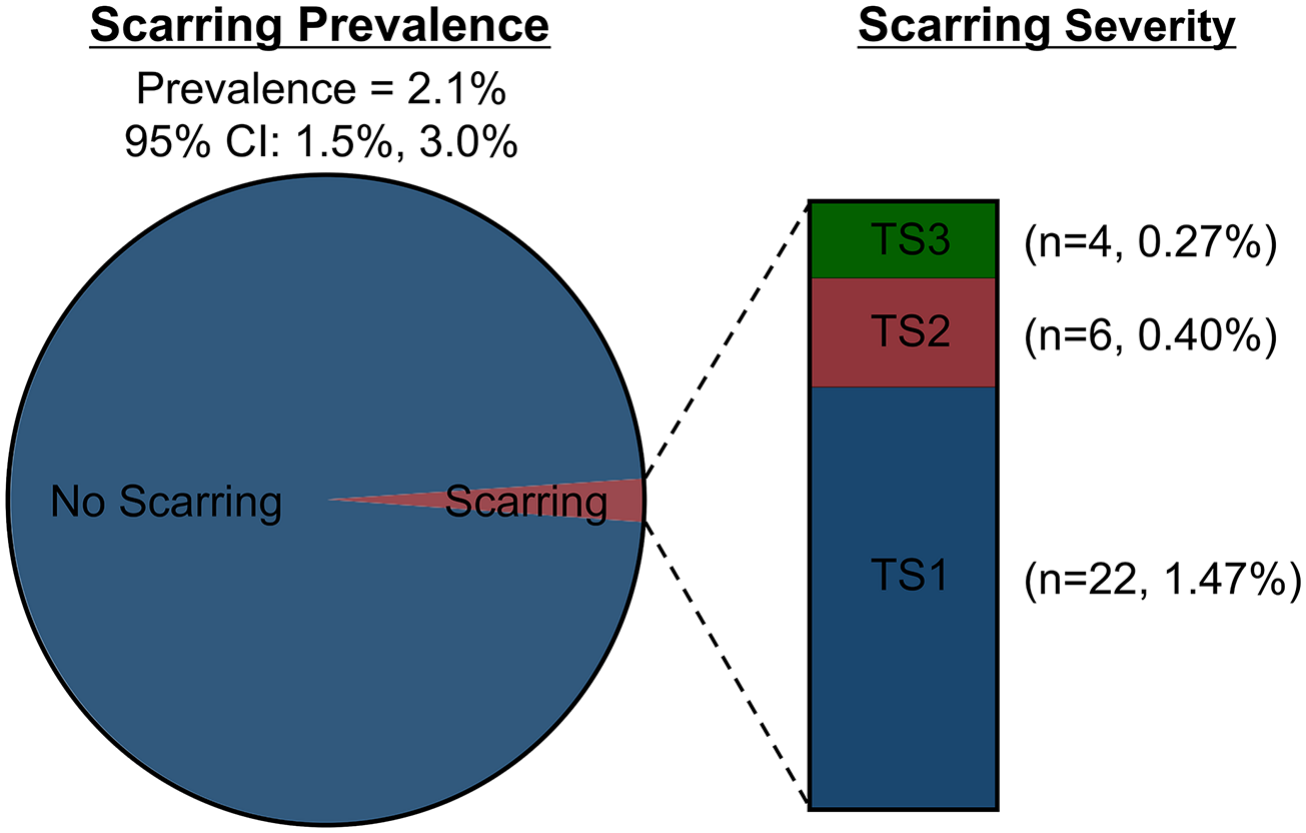
Trachomatous scarring prevalence and severity. Scarring prevalence: *n* = 32/1496 (2.1%, 95% CI: 1.5–3.0%).

**Fig 3:**
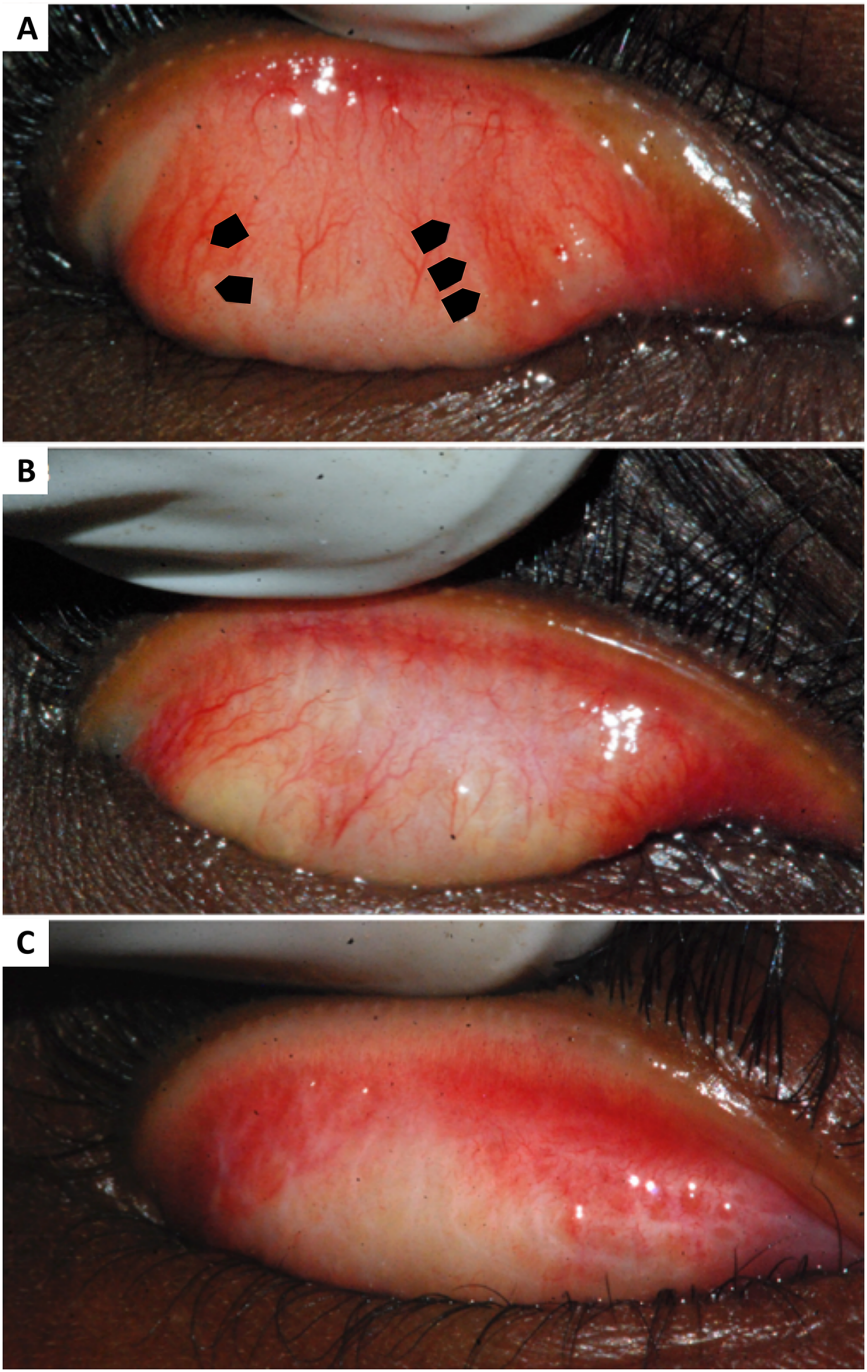
Conjunctival photographs demonstrating trachomatous scarring in children. The black spots in these images are dirt on the lens and not lesions of the conjunctiva. (A) Mild scarring (grade S1) in a six year-old; stellate scars are indicated by arrows on the left, linear scarring is indicated by arrows on the right. (B) Moderate scarring (grade S2) in a five year-old; multiple linear scars are present. (C) Severe scarring (grade S3) in a nine year-old; a meshwork of numerous linear scars, characteristic of trachomatous scarring, is present.

The prevalence of TS was low at 2.1% (95% CI: 1.5%– 3.0%) (Fig 2). When present, TS was usually mild, with 68.8% of cases (22/32 cases) falling into the mildest category, grade S1. Fig 3A–3C illustrates examples of scarring of differing severities that were observed in our study. The prevalence of follicular trachoma and *C*. *trachomatis* infection were 3.2% and 6.5%, respectively, at the time of the survey (Table 2). TS was more prevalent among older children, males, those with active follicular trachoma, and those with *C*. *trachomatis* infection, but none of these associations were statistically significant (*p*-values: 0.14, 0.48, 0.27, 0.15, respectively). The inferences did not change after using a multivariate approach (*p*-values: 0.09, 0.34, 0.59, 0.31).

**Table 2:** Trachomatous scarring (TS) prevalence according to various demographic and clinical characteristics.

Characteristic	TS Absent (*n* = 1,464)	TS Present (*n* = 32)	*p*-value
Age (years), *n* (%)			
1–5	572 (98.6)	8 (1.4)	0.14
6–9	892 (97.4)	24 (2.6)	
Sex, *n* (%)			
Male	714 (97.5)	18 (2.5)	0.48
Female	750 (98.2)	14 (1.8)	
Follicular trachoma, *n* (%)			
Absent	1418 (97.9)	30 (2.1)	0.27
Present	46 (95.3)	2 (4.7)	
*C*. *trachomatis* infection, *n* (%)			
Absent	1371 (98.0)	28 (2.0)	0.15
Present	93 (95.9)	4 (4.1)	

**Fig 4:**
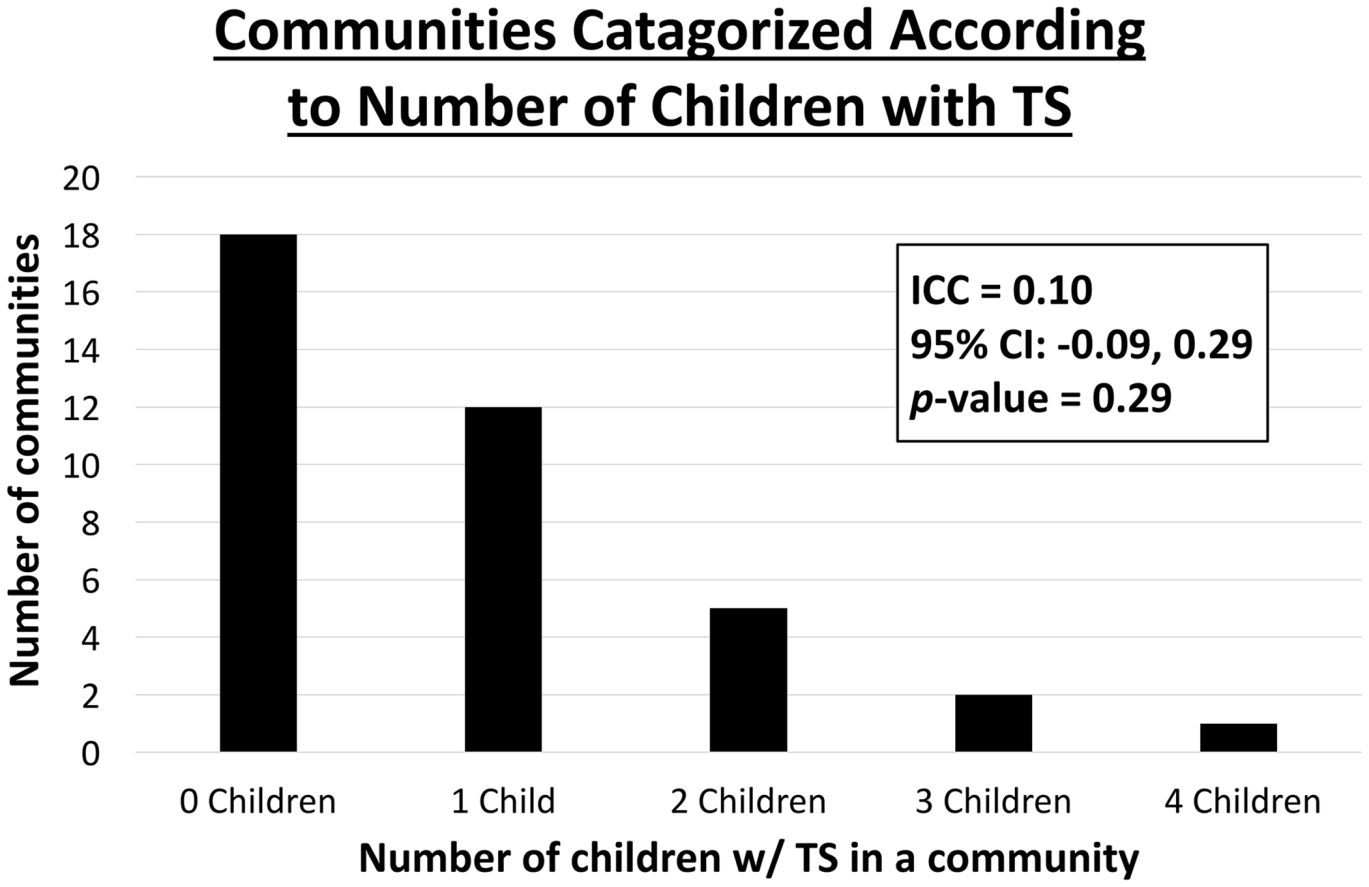
Prevalence of pediatric trachomatous scarring within each community. Abbreviations: trachomatous scarring (TS), intra-class correlation coefficient (ICC), confidence interval (CI).

Nearly half of all communities had no TS present (47.4%, 18/38 communities), and only 21.1% of communities had multiple cases (Fig 4). The most seen in any single community was four cases. The intraclass correlation coefficient was suggestive of an association between TS prevalence and village-level clustering (ICC: 0.10; 95% CI: -0.09–0.29) but did not reach statistical significance (*p*-value: 0.29).

We wanted to assess the relationship between past trachoma prevalence in these communities to the current number of childhood TS cases. We dichotomized communities into having 0–1 TS cases or 2–4 TS cases, and averaged the community prevalence of TF as assessed 3 years prior to the current survey (Table 3). Both follicular trachoma and *C*. *trachomatis* infection were more prevalent in communities with 2–4 TS cases than those with the 0–1 TS cases, but these associations did not achieve statistical significance (*p*-values: 0.08 and 0.17, respectively). Similarly, the prevalence of any active trachoma (follicular trachoma and/or intense trachomatous inflammation) was higher in communities with multiple TS cases, but this was not statistically significant (*p*-value: 0.12).

**Table 3:** Associations between the number of scarring cases and a community’s prevalence of follicular trachoma, any active trachoma, and infection with *C*. *trachomatis*.

Prevalence of:	Village Characteristics		*p*-value*
	0–1 TS Cases (*n* = 30 villages)	2–4 TS Cases (*n* = 8 villages)	
Follicular Trachoma, median (IQR)	4.0% (2.2, 7.5)	6.3% (4.4, 11.2)	0.07
Any active Trachoma**, median (IQR)	4.4% (2.9, 8.3)	8.7% (4.4, 12.6)	0.07
*C*. *trachomatis*, median (IQR)	2.1% (1.0, 3.4)	4.6% (3.7, 5.8)	0.07

Abbreviations: IQR (interquartile range).

*Wilcoxon two-sample test

**Any active trachoma = follicular trachoma and/or trachomatous inflammation

## Discussion

In this large study of children (1,496 participants, 38 communities) we assessed the prevalence and severity of trachomatous scarring among 1–9 year-olds in the formerly hyper-endemic district of Kongwa, Tanzania. The prevalence of TS within this population was low, at 2.1% (95% CI: 1.5%– 3.0%). Scarring was generally mild when present, with 69% of cases falling under the mildest category, grade S1, and no cases falling under the most severe category, grade S4. Our previous work found that children with a constant, severe trachomatous inflammation are at particularly high risk of developing childhood TS. [6] Given the trajectory of decline in trachoma in Kongwa, we now see low prevalence and severity of TS, suggesting that Kongwa’s trachoma elimination efforts may have been particularly effective in decreasing the prevalence of these cases with constant severe inflammation. This contrasts with findings by Ngondi *et al*. in a hyper-endemic area of southern Sudan, in which the prevalence of trachomatous trichiasis was 1.4% among 1–14 year-olds. [15] Trachomatous trichiasis is a consequence of severe scarring from trachoma, and no such cases of trichiasis were seen in our cohort of 1,496 children.

In the aforementioned study by Ngondi *et al*., trichiasis was significantly associated with increasing age, female sex, and the presence of inflammatory trachoma within the household. [15] The association of TS with age and female sex have been demonstrated consistently in numerous studies, and association with sex seems to hold true in all age groups: children, adolescents, and adults. [2,16] It is interesting to note, however, that we found no significant association with these factors in our study, although we did not study scarring in children past the age of 9 years. It is possible that had we included older children and adolescents, a more apparent effect of age would have been seen. The reasons for an increase in scarring with age have to do with cumulative risk of exposure to repeated infection, but clearly, for the age group 1 to 9 years, that has not happened in these communities. It will be fruitful to follow these children longitudinally and observe if scarring rates increase as they age.

Gender differences are observed when females have higher rates of repeated exposures to infection compared to males, but again that appears not to have happened in these communities. While the likelihood of more cases of TS in a community appeared to be associated with greater community prevalence of trachoma as measured three years ago, this is not marked and indeed there was no clustering of cases by community. This is likely due to the fact that even 3 years ago, the community-level prevalence of follicular trachoma rarely exceeded 10%.[13]

It is possible that some of our cases of conjunctival scarring were due to causes other than trachoma, such as adenovirus or trauma, which we would have been unable to distinguish. With such low levels of scarring present, scarring due to other factors might reduce the precision with which we can detect associations between age and gender and scarring due to trachoma. The data did suggest an increase in TS with age, isolation of infection on a swab, and active trachoma, but the associations were not significant. However, the prevalence of conjunctival scarring due to other causes is likely to be less than that due to trachoma in this formerly endemic district.

One limitation of this study was non-participation, which was low at 22%. The most common cause of non-participation was travel outside the village and reflects the highly mobile nature of this population. We found a significant difference with greater latrine ownership in our participants compared to non-participants. This proxy for socio-economic and hygiene status has been linked to greater trachoma prevalence in prior studies[15,17], and suggests that our estimate of scarring may be lower than the true prevalence of scarring in children. However, there was no significant difference between these two groups in the remaining five factors that were assessed, suggesting that participants and non-participants likely were similar populations. Another potential limitation is the fact that the majority of TS was grade S1, which is the most difficult to grade reliably. Our use of multiple graders who had a high inter-rater reliability and were masked to each other’s scores should have increased the reliability of all scoring, including grade S1. This research study used high quality image acquisition and two graders with adjudication to determine scarring. Although we found a low rate of scarring in children, which suggests an important outcome from the reduction of active trachoma, at present the difficulties in assuring quality assessment of scarring in children do not suggest it would be a useful tool for surveillance of trachoma for the global elimination program.

In conclusion, the prevalence of TS among 1–9 years was low in the formerly hyper-endemic region of Kongwa, Tanzania. [14] When present, scarring severity was usually mild. Our findings suggest that the trajectory of decline in trachoma over several years has resulted in a very low risk of scarring for these communities, which is not increasing with age or gender. There is a need for longitudinal follow up to monitor the incidence and progression of scarring in this cohort as they age in communities with low rates of trachoma.

## Supporting information

S1 Data Excel spreadsheet with raw data. (XLSX)

S2 Data STROBE checklist outlining compliance with STROBE criteria for cross-sectional studies. (DOC)
